# Sphingomyelin liposome bearing whole *Leishmania* lysate antigens induce strong Th2 immune response in BALB/c mice 

**DOI:** 10.22038/IJBMS.2020.50471.11496

**Published:** 2021-02

**Authors:** Nazanin Biari, Seyedeh Hoda Alavizadeh, Omid Chavoshian, Azam Abbasi, Zahra Saberi, Seyed Amir Jalali, Ali Khamesipoure, Mahmoud Reza Jaafari, Ali Badiee

**Affiliations:** 1 Nanotechnology Research Center, Pharmaceutical Technology Institute, Mashhad University of Medical Sciences, Mashhad, Iran; 2 Department of Pharmaceutical Nanotechnology, School of Pharmacy, Mashhad University of Medical Sciences, Mashhad, Iran; 3 Department of Immunology, School of Medicine, Shahid Beheshti University of Medical Sciences, Tehran, Iran; 4 Center for Research and Training in Skin Diseases and Leprosy, Tehran University of Medical Sciences, Tehran, Iran; 5 Biotechnology Research Center, Pharmaceutical Technology Institute, Mashhad University of Medical Sciences, Mashhad, Iran

**Keywords:** BALB/c mouse, Humoral immunity, Leishmaniasis vaccines, Liposomes, Phospholipase-A

## Abstract

**Objective(s):**

Whole *Leishmania* lysate antigens (WLL) has been shown to be effective to tackle leishmaniasis in murine models. Although liposomes can be considered as promising vaccines, the activity of phospholipase-A (PLA) in WLL, breeds difficulties to preparing stable liposomal WLL. One strategy to overcome this shortcoming is to use lipids such as sphingomyelin (SM) which is resistant against PLA. This study aim is formulating stable SM liposomes containing WLL and comparing their adjuvant effects with another first generation vaccine , i.e. solube *Leishmania *Antigen (SLA) liposomes in BALB/c mice.

**Materials and Methods:**

BALB/c mice were immunized subcutaneously, three times with 2-week intervals, with Empty-liposome (E-lipo), Particulate WLL, Liposome-WLL, Liposome-SLA and control Buffer, three times every 2-week. Protection was assessed through measuring the swollen footpads and the load of parasites in the spleen. Other factors were used to assess the response of immune system by means of IgG subclasses, IL-4 and IFN-γ levels and intracellular cytokine assay in cultured splenocytes.

**Results:**

Although liposomal WLL were stable in terms of physicochemical properties, mice received Liposome-WLL did not reduce footpad swelling. The load of parasites in spleen and levels of IL-4- were also higher compared to other immunized groups. In terms of IgG isotypes, no considerable difference observed in mice received Liposome-WLL or other formulations.

**Conclusion:**

Liposome-WLL could be a suitable vaccine delivery system when a Th2 response is desired. Also, further studies are warranted to fully understand the role of sphingomyelin in inducing an immune response.

## Introduction

*Leishmania* infections cause a wide range of maladies. Numbers of these illnesses are self-healing such as cutaneous injuries, however, some visceral types for instance, kala-azar, appear to be deadly. Procedures to address these issues have not always been workable. Furthermore, accessible medicines are required to be injected frequently and effective results have been constrained in infected populations (1-3).

For centuries, it was known that recovery from natural infection by cutaneous leishmaniasis (CL) or leishmanization could induce long lasting protection (2). Despite various potentially efficacious antigens and huge efforts to develop vaccines against leishmaniasis, only first generation candidates comprising whole killed parasite or some divisions of *Leishmania* body with or without adjuvants, came into third stage of clinical trials (2, 4-6). The findings in the clinical trials, however, did not meet the requirements primarily on the account of lack of appropriate delivery systems or adjuvant.One possible explanation is that the induced immune response is suboptimal due to the limited Th1 inducer adjuvants for use in human (7). Several pan-antigenic extracts such as ALM (Autoclaved *Leishmania major*), KLM (Killed *Leishmania *major), LAg (*Leishmania *Antigens) and SLA (Soluble *Leishmania* Antigen) have been recently tested for immunization against leishmaniasis, however, most of them did not induce efficient immune responses or resulted in inappropriate immune responses (Th2 immune responses) (8, 9). According to the previous studies, as Th2 or mixed Th2/Th1 response exacerbate leishmaniasis, so, there is a need for proper antigens and delivery systems/adjuvants for inducing pure Th1 responses (10). Today, the number of researches investigating the role of Whole *Leishmania* lysate (WLL) as a combination of protein derivations, genetic contents and phospholipids parts is limited (11-13). Therefore, as WLL is a complex mixture of antigens comprising of all membrane spanning proteins, an array of hydrophobic components and water-soluble proteins, it is more likely to induce desired immunization against cutaneous leishmaniasis in murine models when accompanied by suitable delivery systems and adjuvants.

Nano particulate drug delivery or adjuvant systems have the potential to improve the immune responses against loaded antigen through several mechanisms. Encapsulation of soluble antigens in nanoparticles gives them a particulate nature and enhances their involvements with macrophages as well as antigen presenting cells (APC). Further, nanoparticles could co-encapsulate both antigens and adjuvants and co-deliver them to the same APC (14, 15). Liposomes are lipid-bilayer membranes, that can play a part as an effective depot since they slowly release antigen. These nanoparticles can be considered as safe adjuvant systems with less toxicity which protect antigens from damages, and enhance presentation of the encapsulated antigen by APC through MHC-I or MHC-II pathways (16). 

Egg sphingomyelin (SM) is one of the most commonly used phospholipids for liposome preparation consisting of saturated acyl chains and a single trans double bond in the sphingosine backbone. Sphingolipids are found in the plasma membrane and other associated components such as Golgi and lysosomes of eukaryotic cells (17). Since SM can form strong intermolecular hydrogen bonds with cholesterol (Chol) molecules, it can develop more impermeable membrane compared to similar lipids. Investigations have shown that ciprofloxacin and vincristine liposomes containing SM/Chol are more stable *in vitro* and *in vivo* when compared to other liposomes (18). Inclusion of SM into the membrane of liposome containing cholesterol greatly enhances liposomal stability (19). SM/Chol formulation of vinorelbine showed higher pharmaceutical stability for up to 1 year at 4-8 °C (18).

In a recent experimental study, liposomes containing WLL of *Leishmania major* promastigotes showed to be unstable. Investigations revealed that phospholipase A (PLA) enzyme presents in WLL results in phosphatidylcholine (EPC) breakdown in liposome bilayer and instability. The existence of phospholipase enzyme in *Leishmania* spp. (20) especially in first generation vaccines containing killed *Leishmania* or parasite components seems to be a limitation for preparing an effective liposomal vaccine. Since SM is a lipid without Acyl-bond, in a previous study, it was used to prepare liposome containing SLA, and the results of dynamic light scattering (DLS) did not indicate any sign of SM hydrolysate in chromatogram (21, 22). SM does not contain carboxyl ester bond in its chemical structure and it seems that WLL cannot affect the stability of liposomes containing this lipid (22).

Hence, considering the presence of PLA enzyme in WLL, we aimed at using SM to prepare stable liposome formulations of WLL, as efficient vaccine against challenge with *L. major* promastigotes in BALB/c mice model of leishmaniasis. Thus, a crude extract of detergent solubilized *L. major* promastigotes as a novel antigen was entrapped into liposomes consisting of SM and cholesterol. Liposomes were then characterized for their particle size, surface charge, proteins and phospholipids contents. To investigate the type of immune responses, lesion development, parasite burden in the spleen, cytokine profile, and antibody isotypes were assessed before and after challenge and compared with the control groups.

## Materials and Methods

***Ethics statement***

The protocol was approved by the Institutional Ethical Committee and Research Advisory Committee of Mashhad University of Medical Sciences (Education Office dated March 31,2010; proposal code 88527), based on the Specific National Ethical Guidelines for Biomedical Research issued by the Research and Technology, Deputy of Ministry Of Health and Medical Education (MOHME) of Iran, issued in 2005. Animals were kept in cages and provided with food and water.

This research thesis was carried out according to Mashhad University of Medical Sciences Ethical Committee Acts in Nanotechnology Research Center. *Leishmania major* strain (MRHO/IR/75/ER) used in this experiment is the one used to prepare experimental *Leishmania* vaccine, leishmanin, and leishmanization (7, 23). 

***Animals, parasites, SLA, and WLL***

Female BALB/c mice, 6–8 weeks old, were purchased from Pasteur Institute (Tehran, Iran). The mice were kept in animal house of Pharmaceutical Research Center and fed with tap water and laboratory pellet chow (Khorassan Javane Co., Mashhad, Iran). Animals were maintained in a colony room 12/12 hr light/dark cycle at 21 °C with free access to water and food (7, 23).

Soluble *Leishmania* antigens (SLA) were prepared by using the protocol developed by Scott *et al*. (18) with few changes. Briefly, the parasites harvested at stationary phase were washed 4 times with HEPES-sucrose Buffer (10 mM, 10% w/v, pH 7.4). Then, the number of promastigotes was set to reach 10^6^/ml in Buffer containing enzyme inhibitor cocktail, 50 μl/ml (Sigma, St. Louis, USA). Once parasites have been lysed by freeze-thaw method they were then sonicated in an ice bath. After collecting the supernatant of centrifuged lysate parasites, they were dialyzed against Buffer, sterilized using a 0.22 μm membrane and stored at -70 °C until use. Bicinchoninic acid (BCA) protein assay method was used to determine the concentration of protein (Thermo Scientific, USA).

Regarding WLL, parasites were harvested at stationary phase and were washed 3 times by PBS to remove culture media. Then, the number of promastigotes was adjusted to 10^6^/ml in HEPES buffer (10 mM; pH 7.4) solution consisting 200 mM OEG and was incubated with gentle shaking for 10 min at room temperature. The resulting clear solution containing WLL plus detergent was sterilized using a 0.22 μm membrane. The whole process was performed under the laminar flow hood in sterile microtubes.

***Liposomes preparation and characterization***

To prepare liposome formulations, different molar ratios of sphingomyelin and cholesterol were used as shown in Table 1. As previously mentioned, detergent removal method was used to prepare liposomes (24). Briefly, chloroform in a sterile tube was used to dissolve sphingomyelin (13 μmol/ml; Avanti Polar lipids, USA) and cholesterol (1 μmol/ml; Avanti Polar lipids, USA) as lipid phase. Rotary evaporator (Hettich, Germany) was then used to remove the solvent resulting in a thin layer of lipid film on the wall of tube. The lipid film was then freeze-dried (TAITEC, Japan). The film was dissolved in HEPES-sucrose Buffer (10 mM, 10% w/v, pH 7.4) containing octaethylene glycol monododecyl ether (OEG) 200 mM (Sigma, St. Louis, USA), and WLL (1.5 mg/ml). The detergent-solubilized phospholipids and WLL were mixed and sonicated for 5 min to yield a clear mixed micellar colloid. Liposomes were then formed by detergent removal using SM2 Bio-Beads (BioRad, USA) according to the manufacturer’s instructions. 

The mean diameter and zeta potential of the liposomes were estimated using particle size analyzer (Nano-ZS, Malvern, UK). The concentration of protein entrapped in liposomes was measured by BCA protein assay kit according to the manufacture instructions (Thermo Scientific, USA). The phospholipid content of liposomes was also determined by Bartlett method (58). 

***SDS-PAGE analysis***

Analytical SDS-PAGE was carried out to qualitatively estimate the presence of WLL or SLA in the prepared formulation. The gel consisted of running gel (10.22%, w/v, acrylamide) and stacking gel (4.78%, w/v, acrylamide) at the thickness of 1 mm. The electrophoresis buffer was 25 mM Tris, 192 mM glycine, 0.1% SDS, pH 8.3. Electrophoresis was carried out at 140 V constant voltages for 45 min and after electrophoresis; gels were stained using silver for protein detection.

***Protein concentration measurement by BCA method***

The Pierce BCA Protein Assay includes detergent-compatible formulation based on bicinchoninic acid to detect the color and quantity of total protein. According to the protocol, generally, bovine serum albumin (BSA) is the standard to evaluate the concentration of protein. After pipetting working solution into microplate wells (100 μl/well), standard protein (2 mg/ml) was added to wells (5 or more) and then sample protein was poured (5 μl/well). The added components were thoroughly mixed using pipet. After covering and incubating at 37 °C for 30 min, the plate was then placed at room temperature. To measure the absorbance, standard curve of standard protein was drawn and the data was collected by reading figures in a plate reader at around 562 nm. Finally, sample protein’s concentration was obtained by data of the standard curve. 

***Immunization of BALB/c mice***

Prepared formulations were subcutaneously (SC) injected to mice (10 per group) three times, every 2 weeks: E-lipo (50 μl/mouse), P-WLL (50 μg Particulate WLL/ mouse), Lip-WLL (50 μg WLL/50 μl liposome/mouse), Lip-SLA (50 μg SLA/50 μl liposome/mouse) and Buffer (HEPES 10 mM, sucrose 10% w/v, pH 7.4) alone.

***Footpad swelling measurement with L. major promastigotes***

One week after the injection, the immunized mice (6 mice per group) received *L. major* promastigotes harvested at stationary phase (1×10^6^/ 50 μl/mouse) in the left footpad to measure the expansion of lesions in each mouse footpad by a metric caliper (Mitutoyo Measuring Instruments, Japan). The lesion size was achieved through subtracting the thickness of the uninfected contralateral footpad from that of the infected one.

***Quantitative parasite burden in spleen***

For estimating the counts of parasites in the spleen of mice, limiting dilution assay was used (25). Briefly, 8 weeks after challenge, 3 mice in each group were sacrificed. Their spleens were then homogenized in RPMI 1640 supplemented with 10% v/v heat inactivated FCS (Eurobio, France), 2 mM glutamine, 100 U/ml of penicillin and 100 μg/ml of streptomycin sulfate (RPMI-FCS). The media used to dilute the homogenates in 8 serial 10-fold dilutions. At this point, dilutions were put in each well of flat-bottom 96-well microtiter plates (Nunc, Denmark) which contain solid layer of rabbit blood agar in tetraplicate and incubated at 26±1 °C for 7-10 days. The counts of viable parasites in each sample was calculated based on the highest dilution at which promastigotes could be grown out after the incubation time (26).

***Antibody isotype assay***

Blood samples were collected from 10 mice one week following the last booster injection (before challenge) and 6 mice at week 8 post-challenge. The sera were isolated and kept at -20 °C until being assessed for anti-SLA IgG total, IgG1 and IgG2a antibodies using ELISA method as described before (25). Briefly, 96-well micro titer plates (Nunc, Denmark) were coated with 50 μl of SLA (10 μg/ml in PBS Buffer, pH= 7.3) overnight at 4 °C. Plates were washed and blocked with 200 µl 1% bovine serum albumin in PBS–Tween 20 for 1 hr at 37 °C. Thereafter, serum samples, diluted to 1:200, 1:2000, 1:20000 or 1:200000 with PBS-Tween, were applied to plates. Plates were then treated with HRP-rabbit anti-mouse IgG isotypes according to the manufacturer’s instructions (Invitrogen Inc., USA). Optical density (OD) was read at 450 nm with background subtraction at 630 nm.

***In vitro cytokine production by splenocytes***

ELISPOT assay was performed using mouse ELISPOT kits from U-cytech (Utrecht, The Netherlands) as directed by the supplier. Briefly, at week 8 post-challenge, three mice in each group were sacrificed and splenocytes were and re-stimulated *in vitro* by either mitogen Concanavalin A (Con A) as a positive control or SLA as a recalled antigen. Then, anti-IL-4 or anti-IFN-γ antibodies coated ELISPOT plates were incubated at 4 °C overnight. The splenocytes (5×10^5^ cells/well) were cultured in a total volume of 200 μl in triplicate with DMEM only (as background responses), medium containing Con A (as positive controls), or with medium containing 10 μg/ml of SLA in pre-coated plates. 

After 24 hr (for IFN-γ assay) or 48 hr (for IL-4 assay) incubation at 37 °C, 5% CO_2_, spot counting was done using Kodak 1D image analysate software (Version 3.5, Eastman Kodak, Rochester, USA). The mean number of spots±SD in triplicate wells was calculated and expressed as spot-forming units (SFU) per 10^6^ splenocytes.

***Flow cytometry analysis***

At week 8 post-challenge, pooled splenocytes (10^6^ cells/ml) from 3 mice per group were suspended in medium which contained GolgiPlug^TM^ (1 μl/ml) and stimulated with PMA/Ionomycin cocktail (2 μl/ml) for 4 hr at 37 °C. Following stimulation, 10^5^ splenocytes were incorporated into flow cytometry tubes and washed two times with stain Buffer (2% FCS in PBS). Staining the splenocytes performed by 1 μl anti-CD8a-PE-cy5 antibody and 1 μl anti CD4-PE-cy5 antibody in separate tubes for 30 min at 4 °C. After washing the cells with stain Buffer, they were fixed using Cytofix/CytopermTM solution. Once having been washed twice with Perm/WashTM Buffer, the fixed cells were stained with 1 μl anti-IFN-γ- FITC antibody for 30 min at 4 °C. As regard to CD4 cells, they were also stained with 1 μl anti-IL-4-PE antibody. The cells were washed with Perm/WashTM Buffer and suspended in 300 μl stain Buffer for flow cytometry analysate (BD FACSCalibur™, BD Biosciences, San Jose, USA). 

***Statistical analysis***

One-way ANOVA statistical test was used to assess the significance of the differences among various groups. For significant F value, Tukey–Kramer multiple comparisons test, as a post-test was used to compare the means among various groups of mice. Results with *P*<0.05 were marked as statistically significant.

## Results

***Liposome characterization***

Data in Table 1 shows liposome formulations with different sphingomyelin to cholesterol molar ratios. According to the results of stability, particle size and homogeneity, the molar ratio of 11 to 1 was opted to prepare the final formulations containing WLL (Table 1). The mean diameter and zeta potentials of E-lipo, P-WLL, Lip-WLL and Lip-SLA formulations are shown in Table 2. No significant differences was observed between E-lipo, Lip-WLL and Lip-SLA formulations in terms of particle size and surface charge. The protein and phospholipid concentrations of prepared formulations were measured by BCA and Bartlett method, respectively. The protein concentrations of P-WLL, Lip-WLL and Lip-SLA formulations were not considerably different, however, the amount of phospholipid in Lip-WLL was the highest (Table 2). 

**Figure 1:**
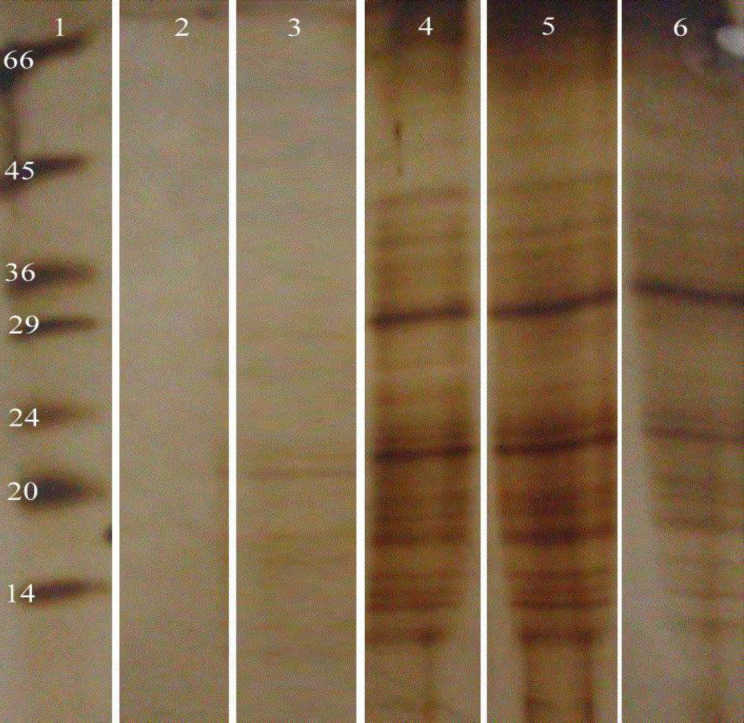
SDS-PAGE analysis of different formulations. Lane 1, low-range protein standard (Sigma); lane 2, Buffer; lane 3, E-lipo; lane 4, P-WLL; lane 5, Lip-WLL; lane 6, Lip-SLA

SDS-PAGE analysis depicted numerous protein bands with different ranges of molecular weight from 10 to 70 kDa. As shown in Figure 1, analysis of liposomes revealed bands corresponding to WLL in lane 3, E-lipo; lane 4, P-WLL; lane 5, Lip-WLL and lane 6, Lip-SLA that confirms the existence of WLL or SLA in all formulations as previously shown (21, 27, 28)

***Challenge results***

**Figure 2:**
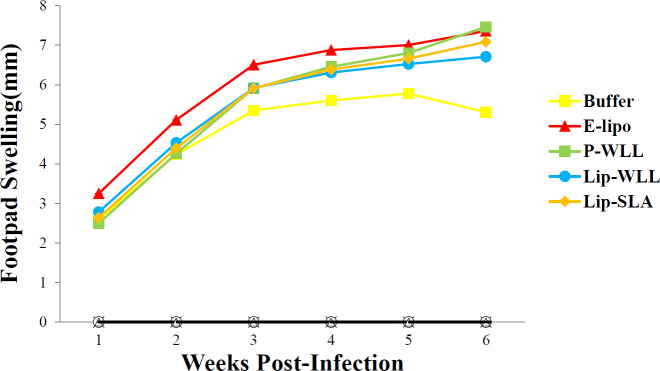
Footpad swelling in BALB/c mice immunized S.C three times in 2-week intervals with Buffer, E-lipo, P-WLL, Lip-WLL and Lip-SLA after challenge with *Leishmania major* promastigotes. One week after the last booster, mice (n=6) challenged in the left footpad with 10^6^ *L. major* promastigotes. Footpad thicknesses were measured during 6 weeks. Each point shows the average increase in footpad thickness±standard error of mean

Progression of lesion was detected weekly by measuring the footpad thickness. The size of lesion expanded at a rapid rate in mice received either liposomal groups or Buffer at week 2 post-challenge. There was no remarkable difference in lesions size of mice immunized between different groups during the observation period (*P*>0.05). Thickness of footpad constantly increased in all groups to reach a plateau after 6 weeks (Figure 2). 

***Splenic parasite burden after challenge***

**Figure 3:**
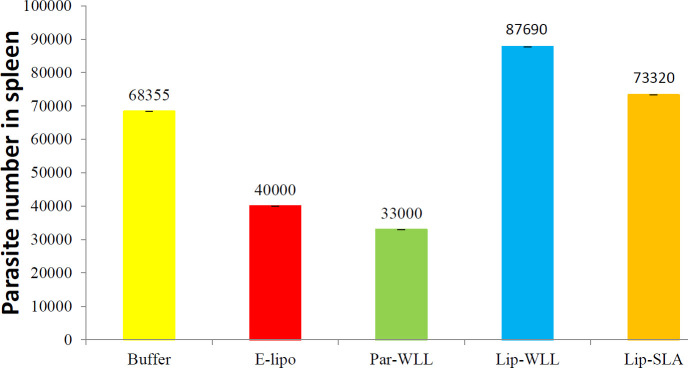
Splenic parasite burden in BALB/c mice immunized Subcutaneous (S.C) three times in 3-week intervals with Buffer, E-lipo, P-WLL, Lip-WLL or Lip-SLA after challenge with *Leishmania major* promastigotes. At 8-week post-challenge, a limiting dilution test was performed on cells isolated from the spleens of individual mice (n=3) and the number of viable parasites per spleen was determined. Each bar represents the average score±SEM (n=3). Significant difference was observed when comparing mice immunized with Lip-WLL or Lip-SLA with those received E-lipo, P-WLL or Buffer (*P*<0.001)

Determination the number of live *L. major *was done in the spleen of mice at week 8 after challenge. All groups of vaccinated mice showed live parasites in their spleen. Splenic parasite loads in mice vaccinated with Lip-WLL or Lip-SLA formulations were considerably greater than P-WLL, E-lip or Buffer (*P*<0.001) (Figure 3).

***Antibody response***

**Figure 4:**
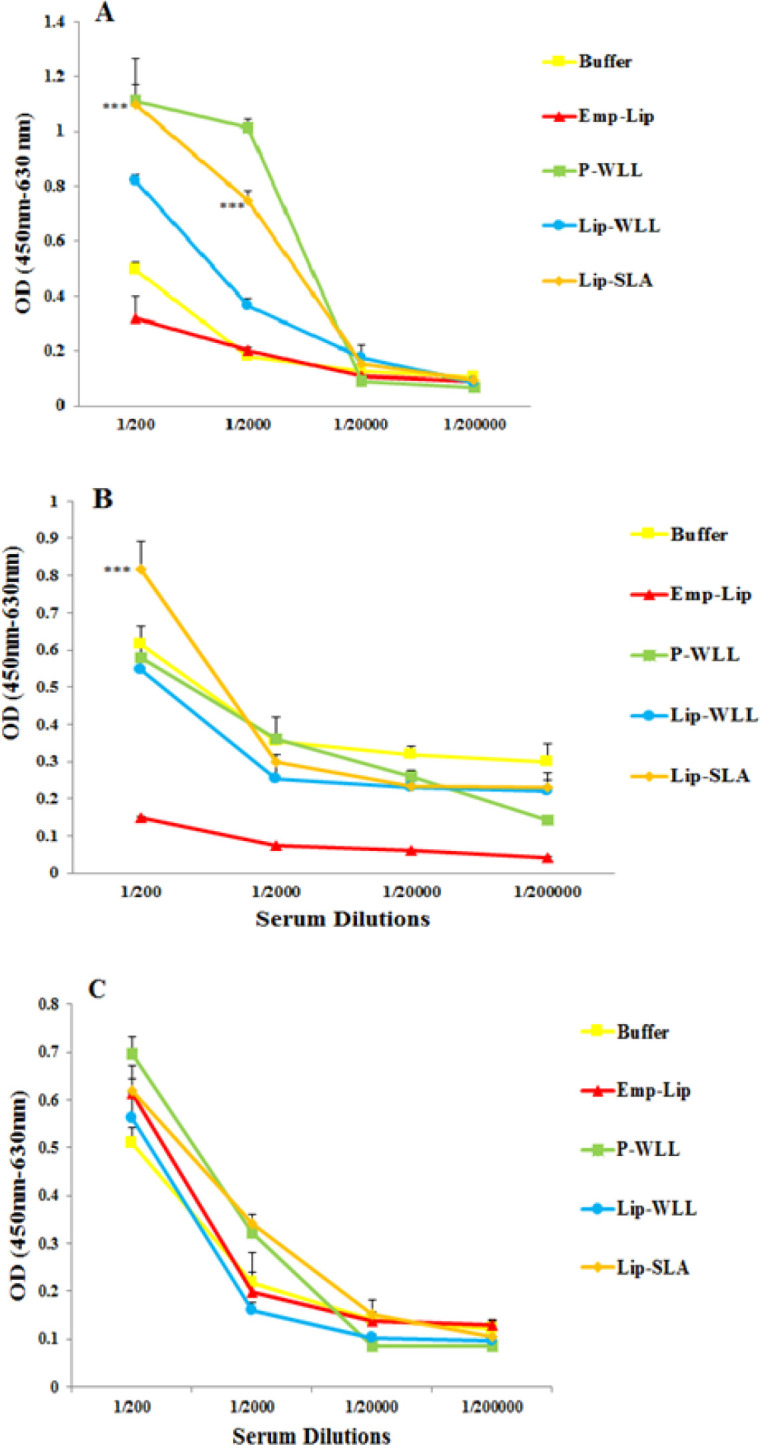
Levels of anti-SLA IgG1 (A), IgG2a (B), and IgG (C) in pooled sera of BALB/c mice immunized SC, three times in 2-week intervals, with E-lipo, P-WLL, Lip-WLL, Lip-SLA or Buffer. Blood samples were collected from mice one week after the last booster and before challenge. The anti-SLA IgG1, IgG2a and IgG levels were assessed using ELISA method. Assays were performed in triplicate at 200, 2,000, 20,000, or 200,000-fold dilutions for each serum sample. Values are significantly higher when mice immunized with Lip-SLA are compared to the other groups in serum dilution of 1/200 (***) indicate *P*<0.001 when comparing different groups and it was not observed any significant difference between Lip-WLL and other groups

**Figure 5:**
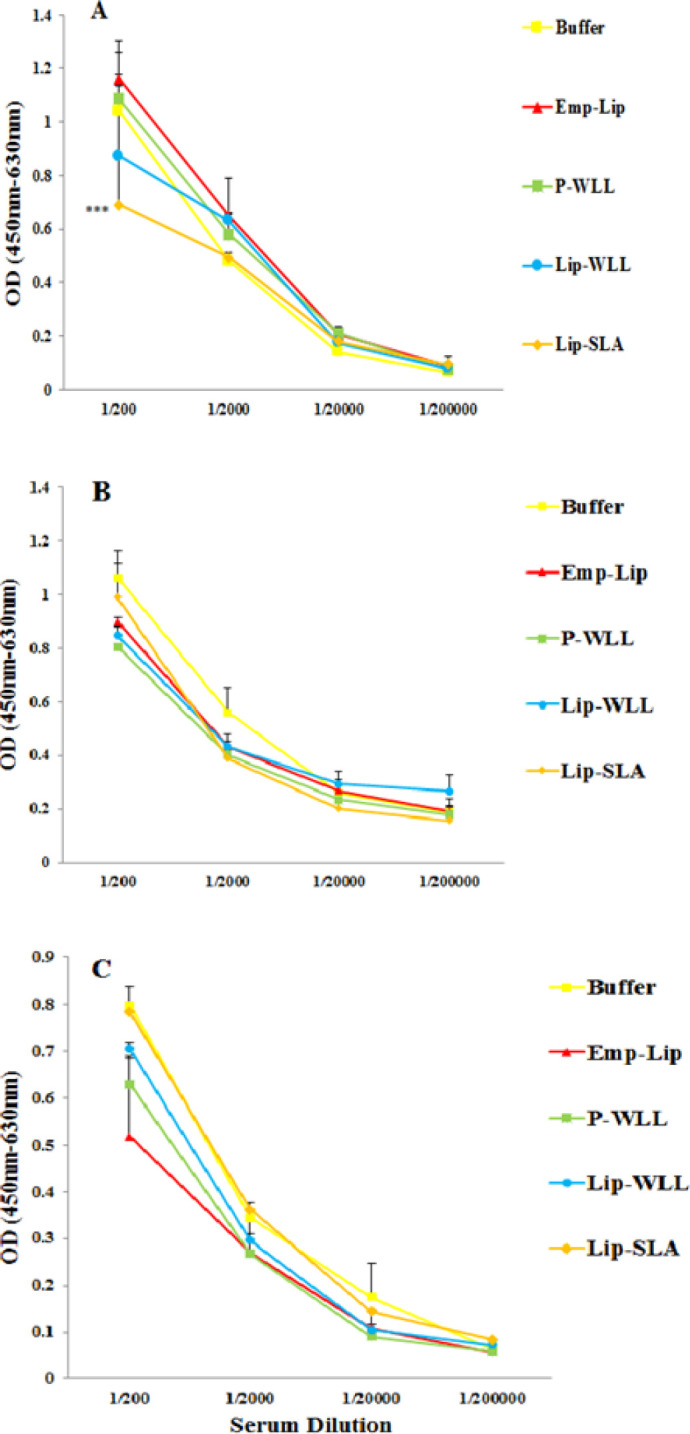
Levels of anti-SLA IgG1(A), IgG2a (B), and IgG (C) in pooled sera of BALB/c mice immunized SC three times in 2-week intervals, with E-lipo, P-WLL, Lip-WLL, Lip-SLA or Buffer. Blood samples were collected from mice 8 weeks after the challenge. The anti-SLA IgG1, IgG2a and IgG levels were assessed using ELISA method. Assays were performed in triplicate at 200, 2,000, 20,000, or 200,000-fold dilutions for each serum sample. Values for IgG1 levels are significantly lower when the mice immunized with Lip-SLA compared to the other groups in serum dilution of 1/200 (***) indicate *P*<0.001 when comparing different groups. There was no significant difference between Lip-WLL and other groups in different dilutions

The type of immune response was determined by titrating anti-SLA IgG antibodies and IgG1 and IgG2a subclasses before (Figure 4A, 4B and 4C) and after (Figure 5A, 5B and 5C) challenge. The sera of pre-challenged mice vaccinated with Lip-SLA or P-WLL indicated significantly higher levels of IgG1 isotypes antibodies compared to E-lipo, Lip-WLL or Buffer (*P*<0.001) in serum dilution of 1:200 and 1:2000. The significant difference was not observed in other dilutions in terms of IgG1. The sera of mice immunized with Lip-SLA showed significantly higher levels of IgG2 antibodies compared to other groups in serum dilution of 1:200 (*P*<0.001). Additionally, the levels of IgG2 in mice received Buffer were generally higher compared to other groups in all dilutions, however the difference was not significant. In terms of IgG, no remarkable difference was observed between mice in different groups before challenge (*P*>0.05).

After challenge, at 1:200 serum dilution, Lip-SLA significantly lowered the level of IgG1 compared to other liposomes or Buffer (*P*<0.001). On the other hand, the level of IgG subtypes in mice immunized with Buffer was totally higher compared to other groups. In general, according to ratio of IgG2a/IgG1, challenge with *L. major* promastigotes increased IgG1, IgG2 and IgG antibody levels in all groups (Table 3).

***In vitro cytokine production by splenocytes***

**Figure 6:**
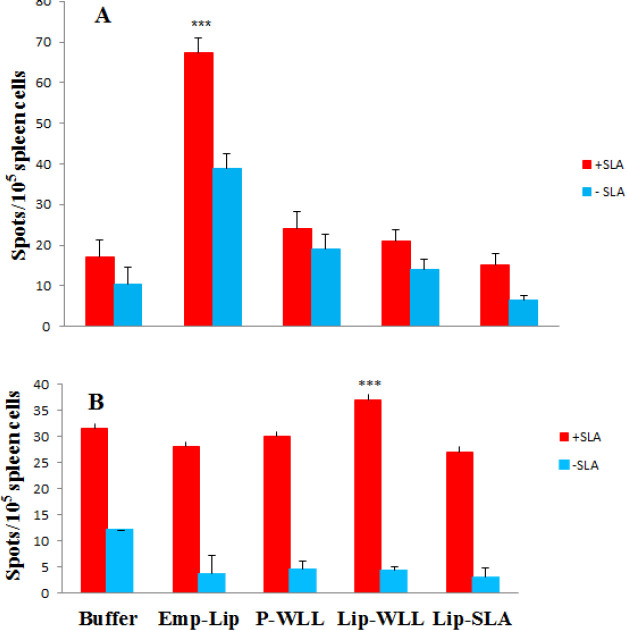
Cytokine levels in mice immunized at week 8 post challenge injection. Mononuclear splenocytes were cultured in the presence of SLA (10 µg/ml) and the level of IFN-γ (A) or IL-4 (B) in the culture supernatants were detected using ELISPOT method. Results are shown as Mean±SEM (n=3). (***) indicate *P*<0.001 when comparing different groups

The supernatants collected from cultured splenocytes at week 8 after challenge were re-stimulated *in vitro* with SLA and analyzed to determine the levels of IFN-γ and IL4, cytokines indicative of Th1 and Th2 responses, respectively. The results of ELISPOT assays indicated that splenocytes isolated from mice immunized with Buffer or Lip-WLL significantly released (*P*<0.001) IFN-γ levels compared to P-WLL, Emp-WLL or Lip-SLA. There was no significant difference in IFN-γ production in mice received Lip-SLA compared with those immunized with P-WLL or Lip-WLL (*P*>0.05), however, the levels of IFN-γ secretion in E-lipo was slightly higher compared to P-WLL or Lip-SLA (Figure 6A).

Although there was no significant difference in IL-4 secretion by splenocytes of mice received either Lip-WLL or P-WLL (Figure 6B), the level of IL-4 detected in splenocytes of mice immunized with Lip-WLL was significantly (*P*<0.001) higher compared to Emp-WLL, Lip-SLA or Buffer groups. 

***Intracellular cytokine assay***

**Figure 7:**
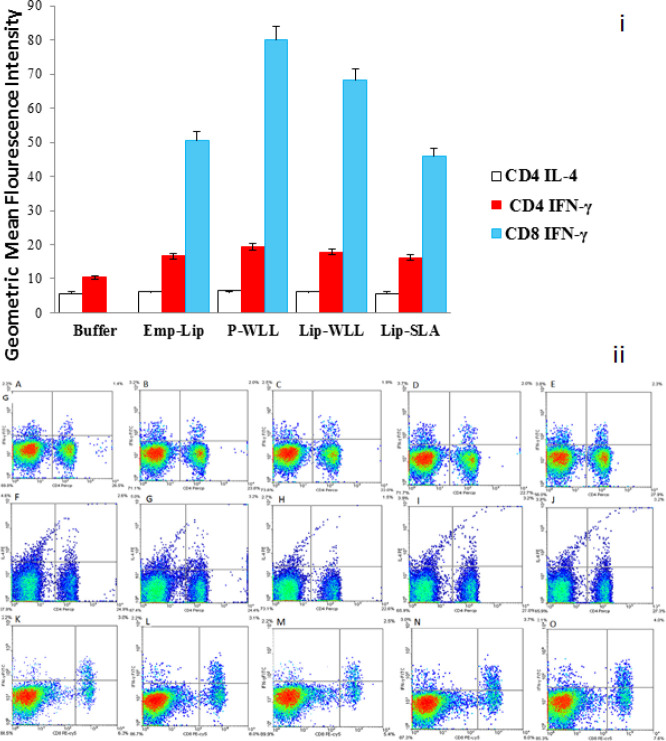
Flow cytometry analysis of geometric mean fluorescence intensity (i) and the mean fluorescence intensity (MFI) (ii) of IFN-γ in gated CD4+ , IL-4 in gated CD4+, and IFN-γ in gated CD8+ for Buffer (A, F, K), E-lipo (B, G, L), P-WLL (C, H, M), Lip-WLL (D, I, N) or Lip-SLA (E, J, O). The results are mean values±SEM of three independent experiments performed in triplicate (n=3)

Flow cytometry analysis using CD8+, CD4+ and IFN-γ markers was also conducted on splenocytes of vaccinated mice at week 8 after challenge. After isolating splenocytes of different groups of immunized mice (3 mice/group), they were stained with PE labeled -CD8 or -CD4 surface markers and then with anti-IFN-γ-FITC or anti-IL-4-PE antibodies, respectively. Results showed the mean±SD (n=3) of geometric mean fluorescence intensity (MFI) level of IFN-γ in gated CD8+ and CD4+ lymphocyte populations and MFI level for IL-4 in gated CD4 s as measured by flow cytometry. Generally, the level of MFI is considerably higher in IFN- γ in gated CD8+ in all groups than two other gates (Figure 7A). Also there is not any notable difference in the amount of IFN-γ in triple gates related to the data of P-WLL, Lip-WLL or Lip-SLA groups, however, the production of mentioned cytokine in P-WLL by CD8+ is remarkably higher than Buffer but not considerably more than other groups (*P*<000.1). On the other hand, there is no difference in the development of IL-4 and IFN-γ by CD4+ in all groups (Figure 7).

## Discussion

Leishmaniasis is caused by disrupted immunological response between the host and the parasite. Thus, cellular immunity with a major role in parasite destruction is one of the most contributing factors to the inhibition of lesion progression. If the parasites growth progress at the beginning of the infection, an imbalanced immune response is elicited, leading to pathological outcome. The resulting inflammatory reactions is responsible for tissue damage and the formation of skin ulcers.

Cutaneous leishmaniasis has long been the emphasis of vaccination, since individuals retrieved from CL evoked by natural contamination or leishmanization, were protected against reinfection and revealed strong immune reactions against *Leishmania* antigens (23, 24, 29) Despite substantial efforts, there is absolutely no promising vaccine available presently to address any form of leishmaniasis issues in human populations (25).

Though the present immunization methodology against leishmaniasis depends on the utilization of recombinant antigens, whole parasite antibodies have the advantages of biochemical arrangement and antigenicity, cost, wellbeing and being utilized in antibody preliminary studies against leishmaniasis (26). Additionally, utilization of entire parasite immunizations resulted in solid cell reactivity (30-32) indicating promising outcomes in rough antigens vaccines (26). One further advantage is that mixed antibodies show a variety of antigenic repertory, prompting the desired immunity (mainly CD4+ and CD8+ IFN-γ-intervened reactions), which is a superior approach compared to puriﬁed subunits antigens or DNA immunizations (26, 33). Broad immunization preliminaries with a mixture of ﬁve slaughtered *Leishmania* stocks or a sole strain of *L. amazonensis* in Brazil and Ecuador, have shown signiﬁcant insurance from characteristic contamination (25). 

Previous studies indicated that protection against Leishmaniasis needs multivalent mixture of antigens including a wide range of protective epitopes which can cover an array of MHC molecules. In the current study, WLL parasite antigens were used due to the presence of plenty of antigen epitopes as well as all integral membrane proteins, water-soluble proteins and hydrophobic components (34, 35). Additionally, sphingomyelin-cholesterol (SM-Chol) nanoliposoms were used since previous studies confirmed the benefits of increased drug loading and release, as well as its resistance to PLA enzyme activity (36). Additionally, the complex of sphingomyelin and cholesterol provides a platform to which various signal molecules are recruited (37). The existence of cholesterol in the vesicles promotes cytoplasmic arrival of the antigens and prohibited lysosomal corruption (38). Expanded *in vivo* stability of the vesicles may additionally improve activation of CD8+ T-cell reaction (39-41). Sphingomyelin (SM), as a major sphingolipid in mammalian cells, together with Chol forms specific lipid micro domains (42, 43).

We have previously used a cationic lipid called DOTAP because the positively charged liposomes could effectively adsorb on the WLL-containing DNA-parasites and the negative-molecules of the antigen-delivering cell. DOTAP has a strong positive charge with a quaternary ammonium moiety at the head group level. Additionally, cationic lipids like DOTAP can stimulate several cellular pathways to activate inflammatory cascades (44). However, the resulting liposomes were not stable due the loss of DOTAP while using Bio-Beads SM adsorbent to remove the detergent (28). In addition, due to two unsaturated bonds in DOTAP fatty acid chains, liposomes prepared with this lipid are unstable *in vivo* (28). Hence, we used sphingomyelin to prepare liposomes. Our hypothesis was confirmed when sphingomyelin was used as an alternative and the resulting liposomes have shown to be stable possibly due to the lack of steroid bond in sphingomyelin structure.

Using sphingomyelin and cholesterol at 11 to 1 molar ratio resulted in stable and homogeneous liposomes with an appropriate particle size. The cholesterol content not only increased the formulation stability, but also prevents lysosomal degradation (38). Besides, in the absence of sphingomyelin and cholesterol lipids (P-WLL), the particles grow larger, which further confirms the role of sphingomyelin in the formation of stable liposomes. 

In several studies, the indispensable role of sphingolipid such as sphingomyelin and its metabolites on the immune system have been shown, either directly or indirectly through the production of several cytokines (45, 46). The role of these compounds in cell growth, differentiation, apoptosis, as well as various inflammatory responses has come to be known as bio-regulators of the cell (36-43). Sphingomyelin metabolism pathway is influenced by the enzyme of the sphingomyelinase (SPMase) that converts it to ceramide which subsequently turns into ceramide-1-phosphate, (C- 1-P) and sphingosine, respectively by enzymes of ceramid kinase and ceramidase (47). Afterwards, sphingosine kinase produces sphingosine-1-phosphate (S-1-P) by adding a phosphate group to sphingosine (48, 49). So far, studies indicated different and important roles of the two metabolites (S-1-P and C-1-P) and their effects on the immune system (20). C-1-P and S-1-P activates phospholipase A2 (PLA2) and cyclooxygenase-2 (COX-2) which form the eicosanoids pathway of inflammatory responses caused by prostaglandins, especially prostaglandin E2 and leukoterian (50-52). Besides, ceramid activates NF- Kb (32), which results in the transcription and induction of over 150 different genes including IL-1B, IL-8, IL-6 cytokines and some inflammatory enzymes such as COX-2 (50, 53). Other studies have shown that sphingolipids generally impedes the production of IL-2, its receptors expression (49, 52, 54, 55) and proliferation of cytokines related to this cytokine (52, 55). IL–2 induce the release of cytokines such as IFN-γ while Th1 + CD4 lymphocyte produce IFN-γ , IL-2, IL-12 and IFN- γ. Sphingomyelin causes an increase in production and expression of IL-4 receptors, which prevents the formation of Th1 + CD4 lymphocytes (50).

The growth rate of the foot ulcer reveals the progression of the disease. Therefore, to compare the immunogenicity of different formulations, the foot size of the mice was measured for six weeks following *L. major *parasite inoculation. As mentioned earlier, no significant difference was observed among various treatment groups. Moreover, according to the mentioned results, the presence of WLL alongside the liposome, aggravated the intensity of swelling which was confirmed by other results as well. The growth and proliferation of the parasite in the spleen of vaccinated mice is another parameter to determine the efficacy of the formulations (56). At week 8 post parasitic injection, the highest parasite load was seen in mice received either Lip-WLL or Lip-SLA which emphasized that Lip-WLL might raise the inflammatory response. The results of cytokine test, 8 weeks after the challenge, demonstrated that splenocytes detached from the mice inoculated with Lip-WLL and E-lipo discharged notably higher measures of IL-4 and IFN-γ, respectively. E-lipo showed the greatest value of IFN-γ and Lip-WLL was not capable of elevating the level of this cytokine and inducing Th1 response. On the other hand, the level of IL-4 secretion by Lip-WLL was remarkably greater than other groups including Buffer, confirming that the co-existence of WLL and sphingomyelin liposome could elevate the signs of leishmaniasis progression. The IgG2a/IgG1 ratio demonstrated that Lip-SLA induced the highest amount of IgG1 (at 1/200, 1/2000) and IgG2a (at 1/200) when compared to other groups one week after challenge, however, 8 weeks after infection induction, the same group showed the lowest level of IgG1 at 1/200 dilutions. Moreover, neither Lip-WLL nor P-WLL induced efficacious immunization against leishmaniasis confirming previous data. On the whole, these data demonstrated the co-existence of Th1/Th2 responses or even a Th2 response with WLL immunization. 

Although estimating the cytokine production develops a useful view, it is not sufficient until some other relative cell subsets like CD4+ and CD8+ T cell are measured. In this study, although P-WLL or Lip-WLL induced considerable IFN- γ by CD8+, none of them were able to produce proper resistant against challenge when compared to all other results. 

As mentioned above, It seems that in our study SM metabolites drive a crucial role in immune response (45, 57). Ceramide itself is considered as a key regulator to activate inflammatory pathway by NF-kβ gene transcription. Ceramide presents a great impression in induction of inflammatory molecules like TNF, IL -6 and IL -8 as well. Contrary to ceramide impact on producing anti-proliferative and pro-apoptic proteins, S1P, another derivation of SM was depicted to emerge protection against ceramide-mediated apoptosis (46). 

Thus, death or survival of the cells is likely associated with mediated equation among these various metabolites. According to the above description, although sphingomyelin contributed to a stable WLL formulation, it apparently results in the induction of a number of cytokines that do not have protection against CL. It appears that sphingomyelin cannot provide the desired immune response to resist leishmaniasis. However, further studies are warranted to fully understand the role of sphingomyelin and other adjuvants in inducing an immune response in *Leishmania* model.

**Table 1:** Mean particle size, poly dispersity index (PDI) and zeta potential of liposome prepared with sphingomyelin and cholesterol at different molar ratios

**Formulations (SM:Chol)**	**Z-average (nm)**	**PDI**	**Zeta Potential (mV)**
20:1	188.7±3.7	0.456±0.63	-21.4±1.2
17:1	143.4±5.2	0.208±0.48	-25.7±1.9
11:1	152.3±5.8	0.229±0.19	-27.7±.16
8:1	380.4±10.2	0.499±0.77	-19.7±1.7
1:0	658.2±23.9	0.548±0.94	-22.1±1.3

**Table 2:** Particle size, poly dispersity index (PDI), zeta potential, protein concentration and phospholipid concentrations of prepared formulations (n=3; Mean±SD). The amount of phospholipid in Lip-SLA was not measured

**Formulations**	**Z-average** **(nm)**	**PDI**	**Zeta Potential** **(mV)**	**Protein Conc.** **(** **µg/ml)**	**Phospholipid Conc. (mM)**
^1^ **E** **-lipo**	116.8 ± 13.8	0.19 ± 0.04	-27.4 ± 6.8	0	6.0 ± 0.61
^2^ **P-WLL**	340.1 ± 10.9	0.55 ± 0.13	-23.5 ± 5.9	856.4 ± 306.5	7.40 ± 0.29
^3^ **Lip-WLL**	143.4 ± 3.2	0.20 ± 0.07	-26.6 ± 10.2	943.1 ± 371.0	12.1 ± 0.28
^4^ **Lip-SLA**	174.4 ± 33.9	0.30 ± 0.13	-25.6 ± 5.7	1009.8 ± 836.1	__

^1 ^Empty-lipoosomes (E-lipo); ^2^ Particulate WLL; ^3 ^Liposome-WLL; ^4^ Liposome-SLA

E-lipo: Empty-lipoosomes; P-WLL: Particulate WLL; Lip-WLL: Liposome-WLL; Lip-SLA: Liposome-SLA

E-lipo: Empty-lipoosomes; P-WLL: Particulate WLL; Lip-WLL: Liposome-WLL; Lip-SLA: Liposome-SLA

E-lipo: Empty-lipoosomes; P-WLL: Particulate WLL; Lip-WLL: Liposome-WLL; Lip-SLA: Liposome-SLA

E-lipo: Empty-lipoosomes; P-WLL: Particulate WLL; Lip-WLL: Liposome-WLL; Lip-SLA: Liposome-SLA

E-lipo: Empty-lipoosomes; P-WLL: Particulate WLL; Lip-WLL: Liposome-WLL; Lip-SLA: Liposome-SLA

E-lipo: Empty-lipoosomes; P-WLL: Particulate WLL; Lip-WLL: Liposome-WLL; Lip-SLA: Liposome-SLA

**Table 3:** The ratio of IgG1/IgG2a before and after challenge at different serum dilutions in different formulations

**Formulation**	**Serum dilutions**							
	**1/200**		**1/2000**		**1/20000**		**1/200000**	
	**before**	**after**	**before**	**After**	**before**	**after**	**before**	**after**
**Buffer**	1.25	1.02	1.67	1.17	2.81	1.83	3.08	2.98
**E-lipo**	0.13	0.75	0.21	0.67	0.41	1.30	0.46	2.22
**P-WLL**	0.53	0.74	0.23	0.69	2.93	1.11	2.16	2.38
**Lip-WLL**	0.66	0.97	0.69	0.68	1.33	1.67	2.61	3.33
**Lip-SLA**	0.74	1.43	0.40	0.78	1.51	1.12	2.54	1.67

E-lipo: Empty-lipoosomes; P-WLL: Particulate WLL; Lip-WLL: Liposome-WLL; Lip-SLA: Liposome-SLA

## Conclusion

Though a stable and nanosized liposome formulation was formulated, based on the results of the footpad lesion, parasitic load, antibody and cytokine assays, Lip-WLL formulation could not induce a significant and effective immune response against *Leishmania major* parasite. Based on the capability to stimulate a Th2 response, further studies are warranted to fully understand the role of sphingomyelin in inducing an immune response.

## References

[1] Croft SL, Sundar S, Fairlamb AH (2006). Drug resistance in leishmaniasis. Clin Microbiol Rev.

[2] Firooz A, Khamesipour A, Ghoorchi MH, Nassiri-Kashani M, Eskandari SE, Khatami A (2006). Imiquimod in combination with meglumine antimoniate for cutaneous leishmaniasis: a randomized assessor-blind controlled trial. Arch Dermatol.

[3] Firooz A, Khatami A, Dowlati Y (2006). Itraconazole in the treatment of cutaneous leishmaniasis. Int J Dermatol.

[4] Khalil EA, El Hassan AM, Zijlstra EE, Mukhtar MM, Ghalib HW, Musa B (2000). Autoclaved Leishmania major vaccine for prevention of visceral leishmaniasis: a randomised, double-blind, BCG-controlled trial in Sudan. Lancet.

[5] Momeni AZ, Jalayer T, Emamjomeh M, Khamesipour A, Zicker F, Ghassemi RL (1999). A randomised, double-blind, controlled trial of a killed major vaccine plus BCG against zoonotic cutaneous leishmaniasis in Iran. Vaccine.

[6] Sharifi I, FeKri AR, Aflatonian MR, Khamesipour A, Nadim A, Mousavi MR (1998). Randomised vaccine trial of single dose of killed Leishmania major plus BCG against anthroponotic cutaneous leishmaniasis in Bam, Iran. Lancet.

[7] Noazin S, Khamesipour A, Moulton LH, Tanner M, Nasseri K, Modabber F (2009). Efficacy of killed whole-parasite vaccines in the prevention of leishmaniasis: a meta-analysis. Vaccine.

[8] Ejazi SA, Ghosh S, Bhattacharyya A, Kamran M, Das S, Bhowmick S (2020). Investigation of the antigenicity and protective efficacy of Leishmania promastigote membrane antigens in search of potential diagnostic and vaccine candidates against visceral Leishmaniasis. Parasit Vectors.

[9] Hojatizade M, Badiee A, Khamesipour A, Jaafari MR (2019). Evaluation of immune response against Leishmaniasis in BALB/c mice immunized with cationic DOTAP/DOPE/CHOL Liposomes containing soluble Leishmania major Antigens. Iran J Parasitol.

[10] Ikeogu NM, Akaluka GN, Edechi CA, Salako ES, Onyilagha C, Barazandeh AF (2020). Leishmania immunity: advancing immunotherapy and vaccine development. Microorganisms.

[11] Afrin F, Rajesh R, Anam K, Gopinath M, Pal S, Ali N (2002). Characterization of Leishmania donovani antigens encapsulated in liposomes that induce protective immunity in BALB/c mice. Infect Immun.

[12] Rivier D, Bovay P, Shah R, Didisheim S, Mauël J (1999). Vaccination against Leishmania major in a CBA mouse model of infection: role of adjuvants and mechanism of protection. Parasite Immunol.

[13] Bhowmick S, Ali N (2009). Identification of novel Leishmania donovani antigens that help define correlates of vaccine-mediated protection in visceral leishmaniasis. PLoS One.

[14] Askarizadeh A, Badiee A, Khamesipour A (2020). Development of nano-carriers for Leishmania vaccine delivery. Expert Opin Drug Deliv.

[15] Zahednezhad F, Saadat M, Valizadeh H, Zakeri-Milani P, Baradaran B (2019). Liposome and immune system interplay: challenges and potentials. J Control Release.

[16] Ravindran R, Maji M, Ali N (2012). Vaccination with liposomal leishmanial antigens adjuvanted with monophosphoryl lipid-trehalose dicorynomycolate (MPL-TDM) confers long-term protection against visceral leishmaniasis through a human administrable route. Mol Pharm.

[17] Barenholz Y (2004). Sphingomyelin and cholesterol: from membrane biophysics and rafts to potential medical applications. Subcell Biochem.

[18] Semple SC, Leone R, Wang J, Leng EC, Klimuk SK, Eisenhardt ML (2005). Optimization and characterization of a sphingomyelin/cholesterol liposome formulation of vinorelbine with promising antitumor activity. J Pharm Sci.

[19] Claassen E, Westerhof Y, Versluis B, Kors N, Schellekens M, van Rooijen N (1988). Effects of chronic injection of sphingomyelin-containing liposomes on lymphoid and non-lymphoid cells in the spleen ransient suppression of marginal zone macrophages. Br J Exp Pathol.

[20] Melendez AJ (2008). Sphingosine kinase signaling in immune cells: potential as novel therapeutic targets. Biochim Biophys Acta.

[21] Chavoshian O, Biari N, Badiee A, Khamesipour A, Abbasi A, Saberi Z (2013). Sphingomyelin Liposomes containing soluble Leishmania major antigens induced strong Th2 immune response in BALB/c Mice. Iran J Basic Med Sci.

[22] Pawlowic MC, Zhang K (2012). Leishmania parasites possess a platelet-activating factor acetylhydrolase important for virulence. Mol Biochem Parasitol.

[23] Rezvan H, Moafi M (2015). An overview on Leishmania vaccines: a narrative review article. Vet Res Forum.

[24] Gillespie PM, Beaumier CM, Strych U, Hayward T, Hotez PJ, Bottazzi ME (2016). Status of vaccine research and development of vaccines for leishmaniasis. Vaccine.

[25] Handman E (2001). Leishmaniasis: current status of vaccine development. Clin Microbiol Rev.

[26] Giunchetti RC, Reis AB, da Silveira-Lemos D, Martins-Filho OA, Corrêa-Oliveira R, Bethony J (2008). Antigenicity of a whole parasite vaccine as promising candidate against canine leishmaniasis. Res Vet Sci.

[27] Firouzmand H, Badiee A, Khamesipour A, Heravi Shargh V, Alavizadeh SH, Abbasi A (2013). Induction of protection against leishmaniasis in susceptible BALB/c mice using simple DOTAP cationic nanoliposomes containing soluble Leishmania antigen (SLA). Acta Trop.

[28] Jafari I, Heravi Shargh V, Shahryari M, Abbasi A, Jaafari MR, Khamesipour A (2018). Cationic liposomes formulated with a novel whole Leishmania lysate (WLL) as a vaccine for leishmaniasis in murine model. Immunobiology.

[29] Costa CH, Peters NC, Maruyama SR, de Brito EC Jr, Santos IK, Working Group on Research Priorities for Development of Leishmaniasis Vaccines (2011). Vaccines for the leishmaniases: proposals for a research agenda. PLoS Negl Trop Dis.

[30] Giunchetti RC, Corrêa-Oliveira R, Martins-Filho OA, Teixeira-Carvalho A, Roatt BM, de Oliveira Aguiar-Soares RD (2007). Immunogenicity of a killed Leishmania vaccine with saponin adjuvant in dogs. Vaccine.

[31] Lasri S, Sahibi H, Sadak A, Jaffe CL, Rhalem A (1999). Immune responses in vaccinated dogs with autoclaved Leishmania major promastigotes. Vet Res.

[32] Mayrink W, Genaro O, Silva JC, da Costa RT, Tafuri WL, Toledo VP (1996). Phase I and II open clinical trials of a vaccine against Leishmania chagasi infections in dogs. Mem Inst Oswaldo Cruz.

[33] Soto M, Ramírez L, Pineda MA, González VM, Entringer PF, de Oliveira CI (2009). Searching genes encoding Leishmania antigens for diagnosis and protection. Scholarly Res Exch.

[34] Moafi M, Rezvan H, Sherkat R, Taleban R (2019). Leishmania vaccines entered in clinical trials: a review of literature. Int J Prev Med.

[35] Saleem K, Khursheed Z, Hano C, Anjum I, Anjum S (2019). Applications of nanomaterials in Leishmaniasis: a focus on recent advances and challenges. Nanomaterials.

[36] Silverman JA, Deitcher SR (2013). Marqibo®(vincristine sulfate liposome injection) improves the pharmacokinetics and pharmacodynamics of vincristine. Cancer Chemother Pharmacol.

[37] Bhat HB, Kishimoto T, Abe M, Makino A, Inaba T, Murate M (2013). Binding of a pleurotolysin ortholog from Pleurotus eryngii to sphingomyelin and cholesterol-rich membrane domains. J Lipid Res.

[38] Hafez IM, Maurer N, Cullis PR (2001). On the mechanism whereby cationic lipids promote intracellular delivery of polynucleic acids. Gene Ther.

[39] Brgles M, Habjanec L, Halassy B, Tomasić J (2009). Liposome fusogenicity and entrapment efficiency of antigen determine the Th1/Th2 bias of antigen-specific immune response. Vaccine.

[40] Copland MJ, Rades T, Davies NM, Baird MA (2005). Lipid based particulate formulations for the delivery of antigen. Immunol Cell Biol.

[41] Ignatius R, Mahnke K, Rivera M, Hong K, Isdell F, Steinman RM (2000). Presentation of proteins encapsulated in sterically stabilized liposomes by dendritic cells initiates CD8(+) T-cell responses in vivo. Blood.

[42] London E (2005). How principles of domain formation in model membranes may explain ambiguities concerning lipid raft formation in cells. Biochim Biophys Acta.

[43] Pralle A, Keller P, Florin EL, Simons K, Hörber JK (2000). Sphingolipid-cholesterol rafts diffuse as small entities in the plasma membrane of mammalian cells. J Cell Biol.

[44] Lonez C, Vandenbranden M, Ruysschaert JM (2008). Cationic liposomal lipids: from gene carriers to cell signaling. Prog Lipid Res.

[45] Ballou LR, Laulederkind SJ, Rosloniec EF, Raghow R (1996). Ceramide signalling and the immune response. Biochim Biophys Acta.

[46] Maceyka M, Spiegel S (2014). Sphingolipid metabolites in inflammatory disease. Nature.

[47] El Alwani M, Wu BX, Obeid LM, Hannun YA (2006). Bioactive sphingolipids in the modulation of the inflammatory response. Pharmacol Ther.

[48] Chalfant CE, Spiegel S (2005). Sphingosine 1-phosphate and ceramide 1-phosphate: expanding roles in cell signaling. J Cell Sci.

[49] Ohanian J, Ohanian V (2001). Sphingolipids in mammalian cell signalling. Cell Mol Life Sci.

[50] Martinova EA (1998). Influence of sphingolipids on T lymphocyte activation. Biochemistry (Mosc).

[51] Pettus BJ, Chalfant CE, Hannun YA (2004). Sphingolipids in inflammation: roles and implications. Curr Mol Med.

[52] Li SY, Chen C, Zhang HQ, Guo HY, Wang H, Wang L (2005). Identification of natural compounds with antiviral activities against SARS-associated coronavirus. Antiviral Res.

[53] Xu Y, Casey G, Mills GB (1995). Effect of lysophospholipids on signaling in the human Jurkat T cell line. J Cell Physiol.

[54] Hauser JM, Buehrer BM, Bell RM (1994). Role of ceramide in mitogenesis induced by exogenous sphingoid bases. J Biol Chem.

[55] Feng Z, Lai Y, Ye H, Huang J, Xi XG, Wu Z (2010). Poly (γ, L-glutamic acid)-cisplatin bioconjugate exhibits potent antitumor activity with low toxicity: a comparative study with clinically used platinum derivatives. Cancer Sci.

[56] Choi CM, Lerner EA (2001). Leishmaniasis as an emerging infection. J Investig Dermatol Symp Proc.

[57] Nixon GF (2009). Sphingolipids in inflammation: pathological implications and potential therapeutic targets. Br J Pharmacol.

[58] Bartlett G R (1959). Phosphorous assay in column chromatography. J Biol Chem.

